# Association between the vaginal microbiome and high-risk human papillomavirus infection in pregnant Chinese women

**DOI:** 10.1186/s12879-019-4279-6

**Published:** 2019-08-01

**Authors:** Yulian Chen, Zubei Hong, Wenjing Wang, Liying Gu, Hua Gao, Lihua Qiu, Wen Di

**Affiliations:** 1 Department of Gynecology and Obstetrics, Renji Hospital, School of Medicine, Shanghai Jiao Tong University, Shanghai, China; 2 Shanghai Key Laboratory of Gynecologic Oncology, Renji Hospital, School of Medicine, Shanghai Jiao Tong University, Shanghai, China; 3 State Key Laboratory of Oncogenes and Related Genes, Shanghai Cancer Institute, Renji Hospital, School of Medicine, Shanghai Jiao Tong University, Shanghai, China

**Keywords:** Pregnancy, Vaginal microbiome, High-risk human papillomavirus

## Abstract

**Background:**

In this study, the association between high-risk human papillomavirus (hrHPV) infection and the vaginal microbiome in pregnant women was evaluated in Chinese cohorts.

**Methods:**

The vaginal bacterial composition of four groups, 38 hrHPV-infected pregnant women (PHR, *n* = 38), pregnant women without HPV infection (PN, *n* = 48), nonpregnant women with hrHPV infection (NPHR, *n* = 19) and nonpregnant women without HPV infection (NPN, *n* = 30), was characterized by deep sequencing of barcoded 16S rRNA gene fragments (V3–4) using Illumina MiSeq.

**Results:**

The results revealed that both pregnancy and HPV infection can increase vaginal bacterial microbial richness and diversity, with the bacterial composition being most influenced by pregnancy. *Lactobacillus* was the most dominant genus among all samples. NPN samples were dominated by CST (community state type) III, mainly composed of *Lactobacillus iners*. Both pregnancy and hrHPV infection were accompanied by an increased proportion of CST I (dominated by *Lactobacillus crispatus*), as opposed to CST III. *Bifidobacterium*, *Bacillus*, *Megasphaera*, *Sneathia*, *Prevotella*, *Gardnerella*, *Fastidiosipila* and *Dialister* were found to be biomarkers for hrHPV-infected women, though different genera (*Bifidobacterium*, *Megasphaera*, *Bacillus*, *Acidovorax*, *Oceanobacillus* and *Lactococcus*) were associated with hrHPV-infected pregnant women.

**Conclusions:**

This work uncovered a probable synergistic effect of hrHPV infection and pregnancy on the vaginal microbial composition. HPV infection in pregnant women was associated with a more complex and diverse microbial environment.

**Electronic supplementary material:**

The online version of this article (10.1186/s12879-019-4279-6) contains supplementary material, which is available to authorized users.

## Background

Human papillomavirus (HPV), a type of DNA virus, is associated with cervical intraepithelial neoplasia (CIN) and cervical adenocarcinoma. Indeed, HPV infection is closely related with cervical cancer and it is also present in other anogenital, head and neck cancers. According to their oncogenic potential, more than 100 types of HPV were classified as high-risk, probable high-risk, and low-risk types [1]. More than 40 types are sexually transmitted and infect the anus and genitals. Compared to warts caused by low-risk HPV types, high-risk HPV types, which account for 99% of cervical neoplasias, are usually asymptomatic [1], and as a result, these latter types are more difficult to detect. Many factors have been proven to increase the risk of genital HPV infection, e.g., sexual behaviors, number of sexual partners, early onset of sexual activity and coinfection with other sexually transmitted infections [2–4].

A large number of microorganisms inhabit the female genital tract, and hundreds of studies have revealed that a dominance of *Lactobacillus* lowers bacterial richness and diversity, indicating a healthy vaginal status. A healthy microbial biofilm may prevent or hinder many urogenital diseases, such as *Candida* infection [5, 6], sexually transmitted diseases [7], urinary tract infections [8, 9] and human immunodeficiency virus (HIV) infection [10]. Some studies to date have also reported that changes in the vaginal microbial structure have a close connection with HPV infection [11–14] and CIN progression [15–17].

Pregnancy is a unique physiological state, and the composition of the vaginal microbiota changes when women become pregnant due to fluctuations in hormone levels [18, 19]. It has been claimed that a vaginal bacterial composition dominated by one or two species of *Lactobacillus* is especially present during pregnancy [18–20]. However, dysbiosis of the vaginal microbiota during pregnancy has been reported to be associated with many complications of pregnancy, such as an increased risk of miscarriage, preterm birth and endometritis [18, 21, 22], yet few studies have investigated the relationship between HPV infection and the vaginal microbiome in pregnant women. It has been reported that children born to HPV-positive mothers have a significantly higher risk for developing infantile anal and genital warts [23], though the occurrence rate of cervical precancerous lesions during pregnancy is comparable to that of nonpregnant women [24]. Overall, the prevalence of HPV in pregnant women remains controversial in the literature, varying from 16.8 to 34.2% [25–28]. Hence, it is still under debate whether pregnant women are predisposed toward HPV infection, and the factors that may influence susceptibility are unclear. Because the vaginal microbiome plays an important role in HPV infection in nonpregnant women, we hypothesized that a different vaginal microbial environment during pregnancy might facilitate HPV infection.

Therefore, the aim of this study is to investigate the association between the vaginal microbial composition and high-risk HPV infection in pregnant women. We attempt to distinguish a different microbial profile of HPV-infected pregnant women from that of nonpregnant women.

## Methods

### Study population and sample collection

Between May 2016 and September 2016, 38 hrHPV (high-risk human papillomavirus)-infected pregnant women (PHR, *n* = 38) and 48 pregnant women without HPV infection (PN, *n* = 48) and with an uncomplicated pregnancy were recruited for this study at their first antenatal care visit at the Department of Obstetrics, Renji Hospital of Shanghai, Jiao Tong University School of Medicine. The inclusion criteria for this cohort study were as follows: age ranging from 25 to 40 years; gestational age between 16 and 30 weeks; and no obvious medical problems or adverse outcomes during any previous pregnancy, such as preterm delivery, diabetes, autoimmune disease or malignant tumors. Participants who had participated in sexual activity or vaginal lavage within 72 h of sampling, reported cervical disease or genital HPV infection, reported vaginal bleeding in the preceding weeks, or used probiotics, antibiotics or corticoids in the preceding 2 weeks were excluded. Two other nonpregnant groups with the same age range and a normal medical history were established as control groups: nonpregnant women with hrHPV infection (NPHR, *n* = 19) and nonpregnant women without HPV infection (NPN, *n* = 30).

All enrolled women were preliminarily screened by both an HPV genotyping test and the ThinPrep cytology test (TCT). HPV detection and genotyping were performed using a commercial HPV genotyping kit for 21 HPV types (Hybribio®, Guangdong): 15 high-risk HPV types (16, 18, 31, 33, 35, 39, 45, 51, 52, 53, 56, 58, 59, 66, 68) and 6 low-risk HPV types (6, 11, 42, 43,44 and 81). Pap smears of cervical exfoliated cells were evaluated by two experienced pathologists. Cytological cell samples were categorized according to Bethesda System criteria [29].

Samples for vaginal microbial analysis were collected from the lateral and posterior fornix using a sterile swab under direct visualization during a speculum examination. The vaginal swab samples were immediately frozen and stored at − 80 °C until extraction.

### Total bacterial genomic DNA extraction and MiSeq sequencing

The swabs obtained were thawed on ice, and cells were suspended in transport buffer by vortexing and transferred to a sterile DNase/RNase-free 2.0 mL tube for enzymatic lysis. The lysate was purified using a QIAamp DNA Mini Kit (Qiagen®) according to the manufacturer’s recommendations. The total genomic DNA quality was assessed by 1% agarose gel electrophoresis, and the DNA concentration was measured using a Nanodrop ND-2000 (Nanodrop®).

The V3–4 hypervariable regions of the 16S rRNA gene were amplified using primers 338F (ACTCCTACGGGAGGCAGCA) and 806R (GGACTACHVGGGTWTCTAAT). An 8-bp barcode sequence was added to the ends of both the forward and reverse primers. Amplification was performed in 50-μl reactions with TransStart Fast Pfu DNA Polymerase (TransGen Biotech®), 200 nM of each primer and 2 μl of template. The reactions were performed using a GeneAmp PCR System 9700 (Applied Biosystems®) under the following thermal profile: 94 °C for 2 min, followed by 30 cycles of 94 °C for 30 s, 57 °C for 30 s, and 72 °C for 30 s, and one cycle of 72 °C for 10 min and a 4 °C hold. Three PCR products per sample were pooled to reduce reaction-level PCR bias. PCR products were examined by 2% agarose gel electrophoresis and then purified using an AxyPrep DNA Gel Extraction Kit (AXYGEN®). Amplicons were quantified using the QuantiFluor-ST™ system (Promega®). All sequencing was performed using the Illumina MiSeq platform at Majorbio Biopharm Technology Company (Shanghai).

### Sequence analysis

Low-quality sequences with an average quality score less than 20, a length shorter than 50 bp, or any mismatches to the primers or barcode containing chimeras were removed by Trimmomatic [30]. Operational taxonomic units (OTUs) were defined using a cutoff value of 97% by QIIME. The taxonomy of OTUs (from phylum to species) was determined using the Ribosomal Database Project (RDP) classifier script (version 2.2). A Silva database was also used in this study (Release 128 http://www.arb-silva.de). Chimeric sequences and singletons were removed prior to taxonomic assignments.

The Chao richness estimator and Shannon alpha-diversity index were calculated using mothur (version v.1.30.1) [31]. Comparisons between different groups were assessed by Student’s t-test. Based on unweighted UniFrac distances calculated by the vegan package implemented in R [32], principal coordinates analysis (PCoA) was used to assess the difference in overall microbial community composition for beta-diversity assessment among four groups using R and tested by ANOSIM test. Taxonomic differences between the four groups were analyzed using the nonparametric Kruskal-Wallis test at different levels. Microbiological markers were detected using the linear discriminant analysis (LDA) effect size (LEfSe) algorithm [33]. To conform to vaginal community state types (CSTs) [34], hierarchical clustering analysis was conducted based on Jensen–Shannon distances between all pairs of community states and Ward linkage methods, as previously published [35]. Redundancy analysis (RDA) was applied to evaluate correlation between specific taxa and HPV infection or pregnancy using Canoco [36]. Heatmaps were generated and statistical analyses performed using R. A *p*-value < 0.05 was considered significant in all statistical analyses mentioned above.

## Results

### Characteristics of the study population

The average ages of each group (PHR, PN, NPHR, NPN) were 30.13 ± were, 29.77 ± 29., 33.53 ± 364 and 34.90 ± 4.17 years, respectively. The main genotype of HPV in both pregnant and nonpregnant women was HPV-16 (Additional file 1: Table S1 and Additional file 2: Table S2). All cytological tests were normal, and no lesions were detected. The demographic characteristics of each group are presented in the Additional file 3: Table S3).

### Sequencing results

After filtering low-quality reads, 3,968,879 assembled clean reads were obtained from 135 samples, with a mean read length of 447.37 ± 7n57 bp. For normalization, the reads in each sample were randomly subsampled to the lowest number of 20,308 in sample NP_24 (PN group). After removing singletons (the OTUs contained less than 2 reads), 320 OTUs were identified, ranging from 10 OTUs in sample 4398 (NPN group) to 199 OTUs in sample 142_HH (PHR group) (Additional file 4: Table S4).

### Vaginal microbiota richness and diversity

**Fig. 1:**
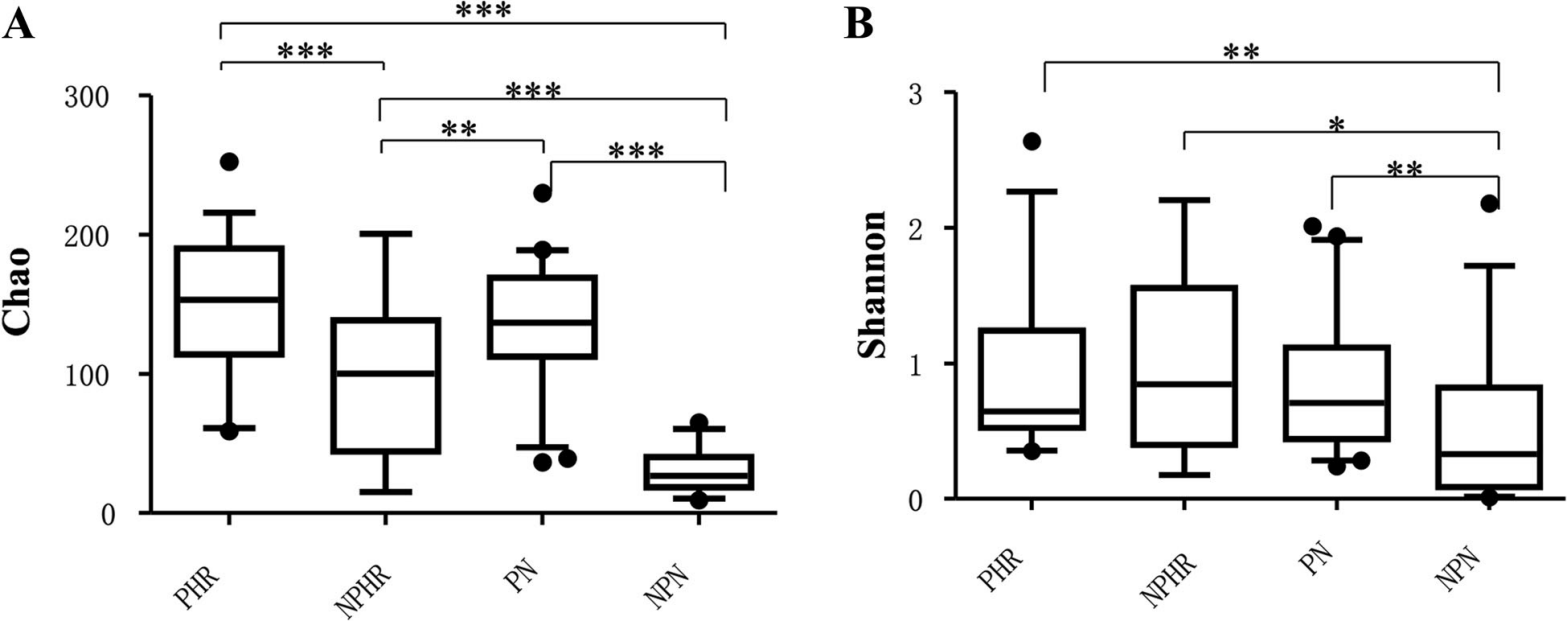
Vaginal bacterial richness and diversity in four groups. **a** Chao index; **b** Shannon index; Student’s t-test was used to compare differences between two groups; data are presented as the mean ± SD; ^***^: *p* ≤ 0.001; ^**^: 0.001 < *p* < 0.01; *: *p* < 0.05

At the OTU level, microbial richness and diversity were estimated using Chao and Shannon indices, respectively, as shown in Fig. 1. These two indices revealed that both pregnancy and HPV infection increased vaginal bacterial richness and diversity. The means of Chao and Shannon indices were much higher in groups PN (135.97 ± 7ere and 0.82 ± and, respectively) and PHR (151.24 ± (151. and 0.96 ± and, respectively) than in NPHR (94.20 ± 094sp and 0.91 ± and, respectively) and NPN (29.94 ± (29. and 0.51 ± 1nd, respectively). Furthermore, the influence of pregnancy on bacterial richness was greater than that of HPV infection (Chao index, PN vs. NPHR: 135.97 ± N vs. > 94.20 ± 4.20., *p* = 0.002 < 0.01).

### Vaginal bacterial structure and beta-diversity in different groups

**Fig. 2:**
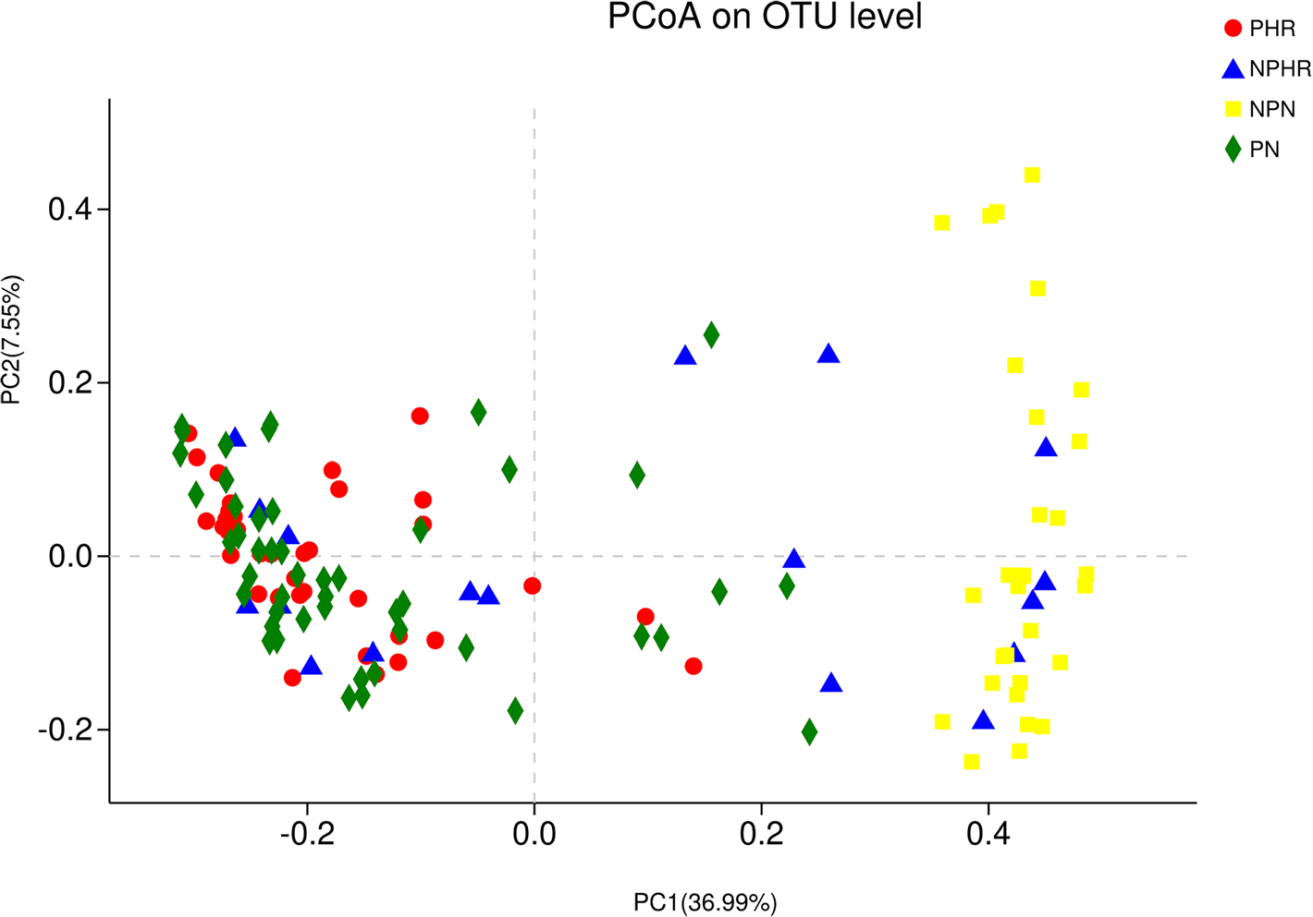
Unweighted UniFrac principal coordinates analysis (PCoA) plot comparing sample distribution for the different groups

In PCoA, the first two principal components explained 36.99 and 7.55%, respectively, of the variance along the first and second axes, with the PHR, PN and NPHR samples visually separated from the NPN sample (Fig. 2). Comparison between two groups based on the ANOSIM test revealed that the bacterial structure of groups PHR, PN, NPHR and NPN were significantly different from each other, except for PHR vs. PN (*R* = − 0.0062, *p* = 0.545) (Additional file 7: Table S5).

### Taxonomy of the vaginal microbiota in different groups

**Fig. 3:**
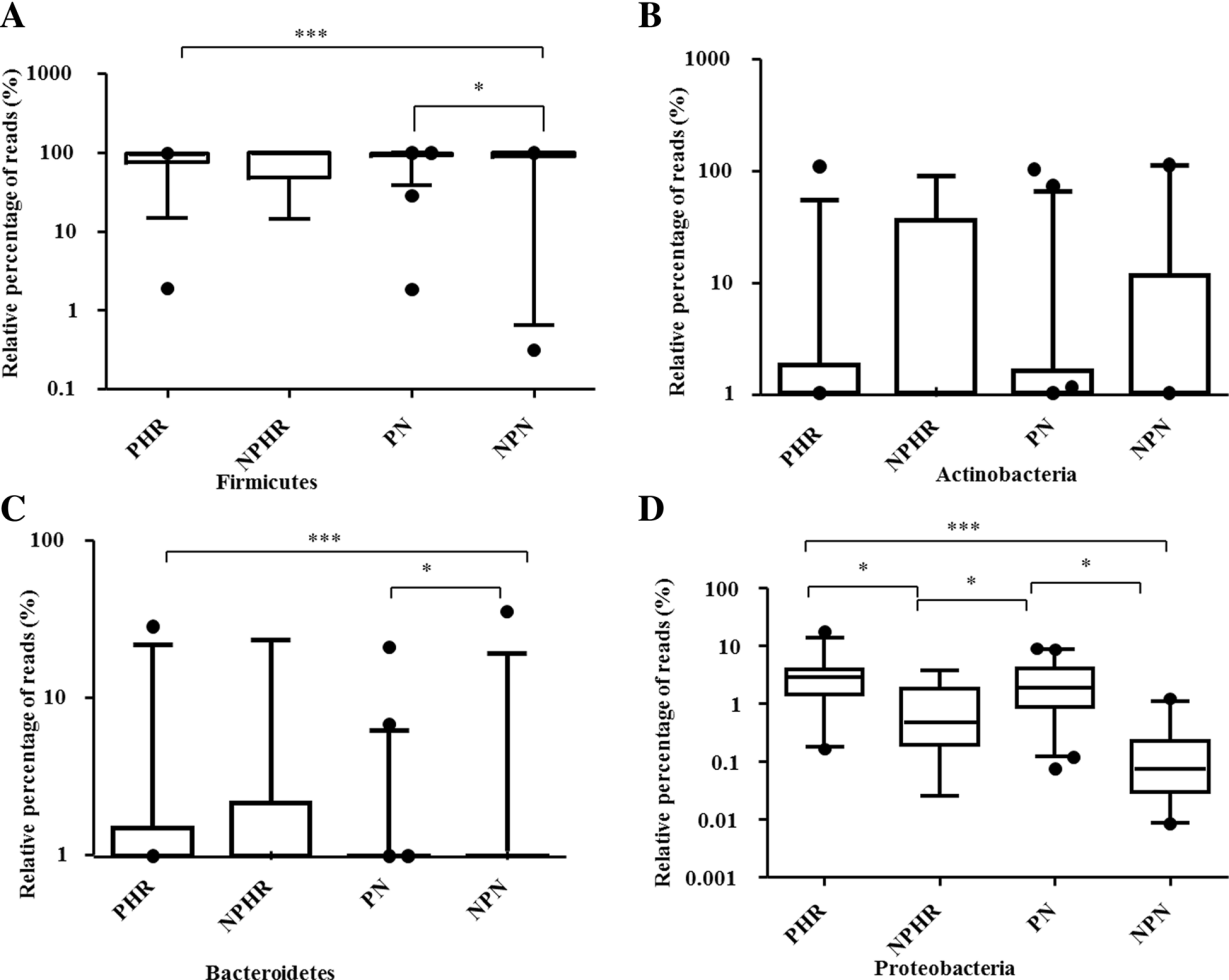
Relative abundance counts of Firmicutes (**a**), Actinobacteria (**b**), Bacteroidetes (**c**) and Proteobacteria (**d**), which were found to be the most abundant phyla across all samples. The Wilcoxon test was used to compare differences in the abundance of each phylum between two groups. ^***^: *p* ≤ 0.001; ^**^: 0.001 < *p* < 0.01; *: *p* < 0.05

Overall, 22 bacterial phyla were recovered across all samples (Additional file 5: Figure S1), and 99% of the samples were dominated by Firmicutes, Actinobacteria, Bacteroidetes and Proteobacteria (Additional file 5: Figure S1). Firmicutes was the most abundant phylum, accounting for 85.57, 78.32, 88.73, and 82.94% of the NPN, NPHR, PN and PHR groups, respectively (Fig. 3a). Furthermore, pregnancy tended to increase the proportion of Firmicutes (PHR > NPN, PN > NPN, *p* < 0.05), but no influence was found for HPV infection (NPN vs. NPHR, *p* > 0.05). There were no differences among the different groups with regard to the proportion of Actinobacteria (Fig. 3b). The percentage of Bacteroidetes was significantly higher in the PHR group (3.06%) than in the NPN group (1.40%) but was lower in the PN group (1.10%) (Fig. 3c). Moreover, the proportions of Proteobacteria (Fig. 3d) were significantly increased after pregnancy, from 0.19% in the NPN group to 3.04% in the PHR group and 2.61% in the PN group (*p* < 0.05). HPV infection also decreased the relative percentage of Proteobacteria, from 2.61% in the PN group to 0.98% in the NPHR group (*p* < 0.05), though HPV infection did not change the dynamics of Proteobacteria during pregnancy (PHR vs. PN, *p* > 0.05).

**Fig. 4:**
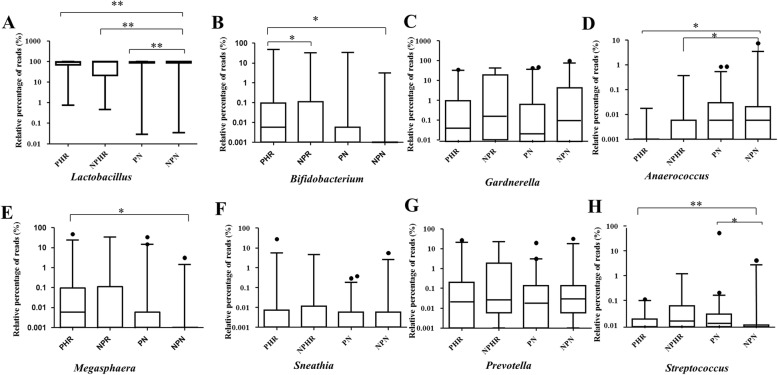
Relative abundance counts of *Lactobacillus* (**a**), *Bifidobacterium* (**b**), *Gardnerella* (**c**), *Anaerococcus* (**d**), *Megasphaera* (**e**), *Sneathia* (**f**), *Prevotella* (**g**) and *Streptococcus* (**h**), which were found to be the most abundant genera across all samples. The Wilcoxon test was used to compare differences in the abundance of each phylum between two groups. ^***^: *p* ≤ 0.001; ^**^: 0.001 < *p* < 0.01; *: *p* < 0.05

At the genus level, a total of 192 taxa were found across all samples (Additional file 6: Figure S2), with *Lactobacillus* being the most dominant genus overall. In addition, the proportion of *Lactobacillus* was significantly reduced during pregnancy and HPV infection compared to the NPN group (PHR: 75.27%, PN: 81.30%, NPHR: 70.10%, NPN: 84.58%) (Fig. 4a). In addition, pregnancy had a negative effect on the abundance of *Bifidobacterium*, which sharply decreased from 4.37% in the NPHR group to 0.02% in the PHR group (*p* < 0.05) (Fig. 4b). Pregnancy, however, increased the relative percentage of *Streptococcus* (Fig. 4h) from 0.19% in the NPN group to 1.11% in the PN group (*p* < 0.05). It was surprising to find that the abundance of *Bifidobacterium* was increased in HPV-infected patients (from 1.72% in the NPN group to 4.37% in the NPHR group, *p* < 0.01) (Fig. 4b). Conversely, HPV infection had a negative effect on the abundance of *Anaerococcus,* though pregnancy did not influence its proportions (NPHR: 0.04%, PHR: 0.002% vs. NPN: 0.27%, *p* < 0.05) (Fig. 4d). *Megasphaera* was significantly more abundant in the PHR group (3.74%) than in the NPN group (0.10%) (Fig. 4e), which revealed that the association between pregnancy and HPV infection increased the proportion of *Megasphaera*. The dual effect of pregnancy and HPV infection (PHR: 0.01% vs. NPN: 0.19%) also influenced the abundance of *Streptococcus* (Fig. 4h).

### Identification of vaginal microbiological markers in different groups

**Fig. 5:**
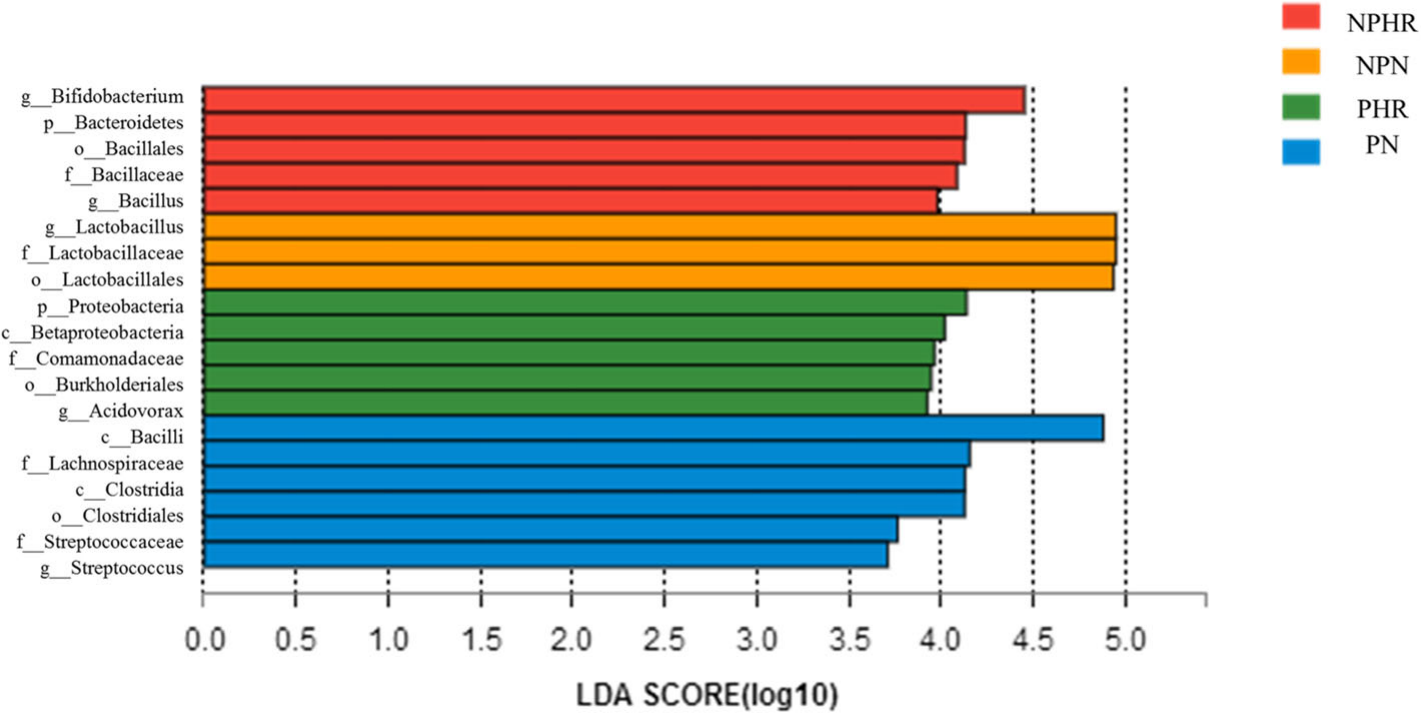
The unique taxa and microbiomarkers for different groups. Shown is a histogram of LDA scores computed for features differentially abundant in the four groups

LEfSe modeling was employed to identify microbiological markers related to HPV infection and pregnancy (Fig. 5). The threshold for the logarithmic LDA model score for discriminative features in this study was 3.5. The most abundant genus in the NPN group was *Lactobacillus*. HPV infection was strongly associated with two genera, *Bifidobacterium* and *Bacillus*. *Streptococcus* at the genus level, Lachnospiraceae at the family level, and Clostridiales at the order level were three taxa related to pregnancy. Nevertheless, the PHR group was associated with *Acidovorax* and members of the families Comamonadaceae, both from the phylum Proteobacteria.

### Characteristics of vaginal community state types (CSTs) for different groups

**Fig. 6:**
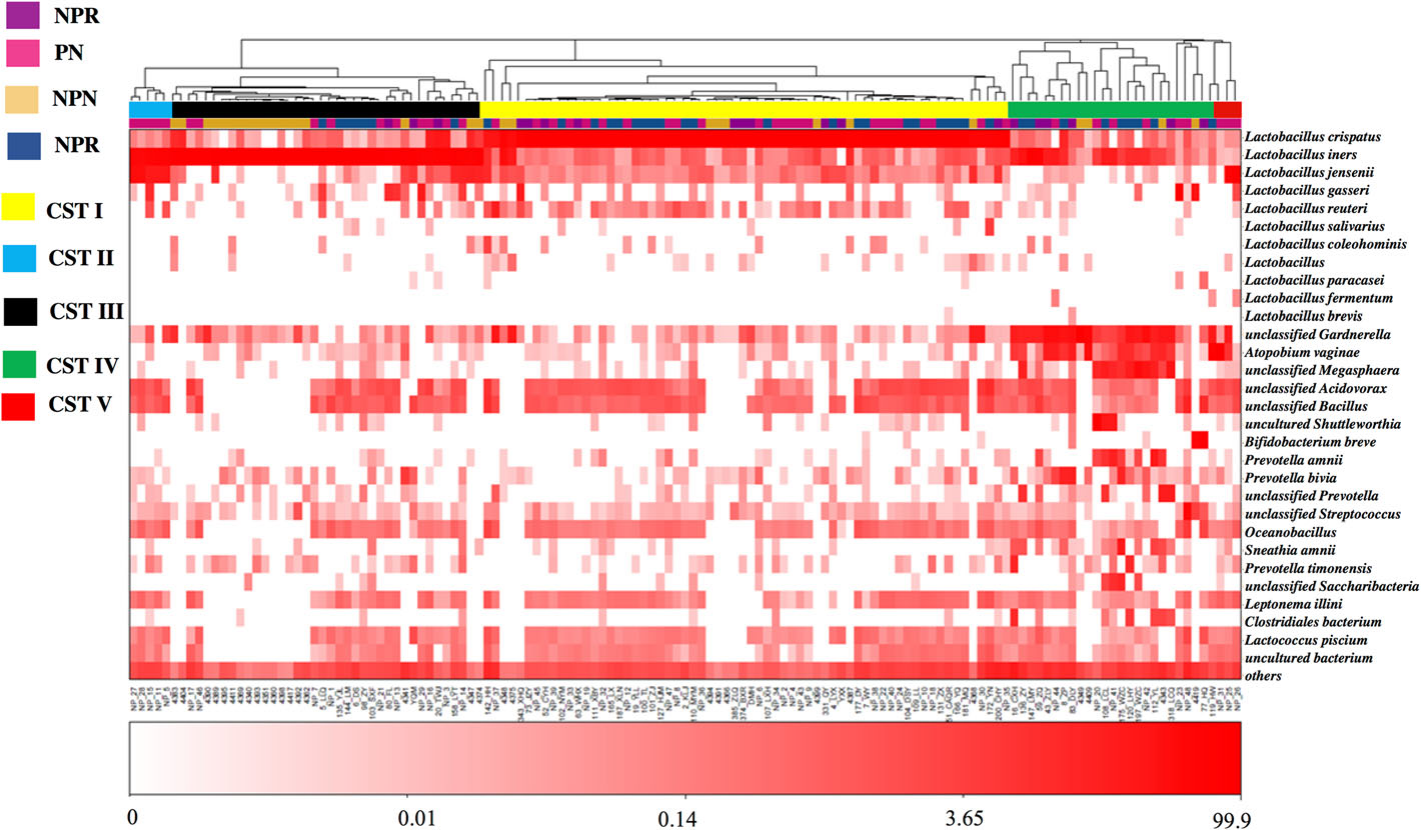
Heat map of the relative abundance of the 31 most abundant bacterial taxa found in the vaginal bacterial communities of all participants in the study. Ward linkage clustering was used to cluster samples based on their Jensen-Shannon distance. Identified CSTs are labeled as I, II, III, IV and V, according to a previous naming convention

The vaginal bacterial CST analysis visualized by hierarchical clustering revealed that all samples clustered into five major groups: CST I, CST II, CSTII, CST IV and CST V (Fig. 6). The most commonly observed community was CST I (64/135, 47.4%), followed by CST III (38/135, 28.1%), CST IV (26/135, 19.3%), CST II (2/135, 1.5%) and CST V (5/135, 3.7%). The proportions of CSTs in different groups are shown in Table 1. Samples in the NPN group were assigned to CST III (18/30, 60.0%). Pregnancy converted the vaginal bacterial community structure from CST III to CST I, as the PN group was dominated by CST I (24/48, 50.0%) and presented less CST III (10/48, 20.8%). Similarly, the proportion of CST I increased to 52.6% (10/19) in the NPHR group. CST I was also dominant in hrHPV-infected pregnant women (group PHR) (22/38, 57.9%), and hrHPV infection increased the proportions of CST IV in both pregnant and nonpregnant women, as NPHR (31.6%) > PHR (23.7%) > PN (14.6%) > NPN (13.3%).

**Table 1:** The distribution of community state types (CSTs) in different groups

	PN N (%)	PHR N (%)	NPN N (%)	NPHR N (%)
CST I	24 (50.00)	22 (57.90)	8 (26.70)	10 (52.60)
CST II	2 (4.20)	0 (0)	0 (0)	0 (0)
CST III	10 (20.80)	7 (18.40)	18 (60.00)	3 (15.80)
CST IV	7 (14.60)	9 (23.70)	4 (13.30)	6 (31.60)
CST V	5 (10.40)	0 (0)	0 (0)	0 (0)

### Redundancy analysis of samples

**Fig. 7:**
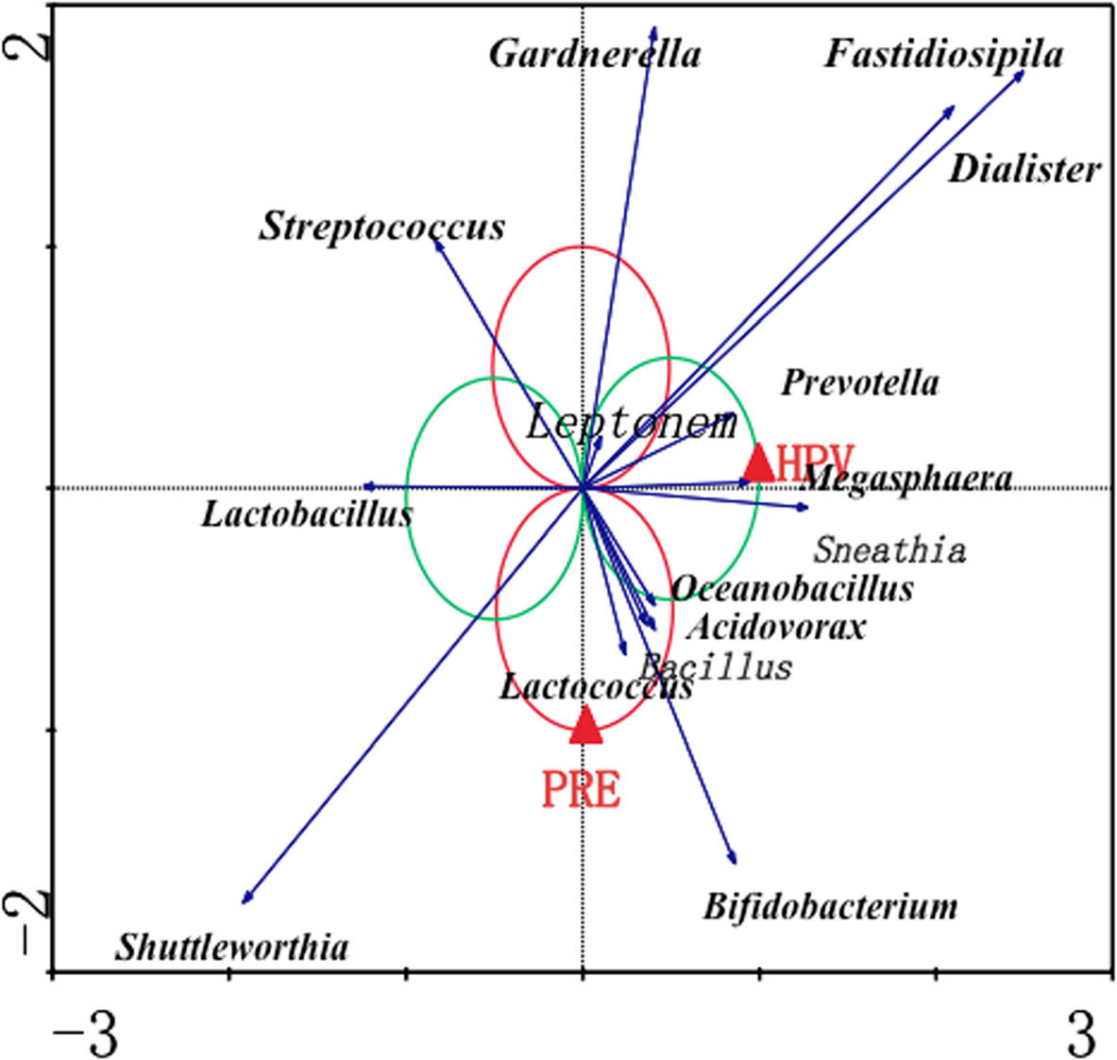
Redundancy analysis of correlations between different specific genera and HPV infection or pregnancy

The results of RDA (Fig. 7) showed that the abundances of *Megasphaera*, *Sneathia*, *Prevotella*, *Gardnerella*, *Fastidiosipila* and *Dialister* correlated positively with HPV infection; in contrast, the abundances of *Lactobacillus*, *Streptococcus* and *Shuttleworthia* correlated negatively with HPV infection. In addition, the abundance of *Shuttleworthia* correlated positively with pregnancy, and that of *Gardnerella* correlated negatively with pregnancy. The abundances of *Bacillus*, *Acidovorax*, *Oceanobacillus*, *Lactococcus* and *Bifidobacterium* correlated positively with a dual influence of pregnancy and HPV infection.

## Discussion

Our study addressed an undetermined topic about the association between the vaginal microbiota and hrHPV infection in pregnant women. We observed that both pregnancy and hrHPV infection were accompanied by increased vaginal bacterial richness and diversity. *Lactobacillus* was still the most abundant genus in all groups; however, both hrHPV infection and pregnancy had a negative influence on its abundance. Pregnancy and hrHPV infection were also accompanied by an increased proportion of CST I (dominated by *Lactobacillus crispatus*), as opposed to CST III. The abundances of various genera were differentially influenced by hrHPV infection and pregnancy. Overall, more anaerobic bacteria were associated with hrHPV infection and pregnancy.

According to our results, pregnancy increased vaginal bacterial richness and diversity, though some previous studies have reported lower vaginal microbial diversity during pregnancy [19, 20, 37]. A possible reason for the different findings might be that higher estrogen concentrations during pregnancy resulting in an accumulation of glycogen metabolized to lactic acid by *Lactobacillus* spp. on the upper layer of the vaginal epithelium, and leading to an increased abundance of *Lactobacillus* spp. in the vagina, thus decreasing overall bacterial richness and diversity. In this study, we observed a reduced abundance of *Lactobacillus* during pregnancy, which might lead to the growth of other bacteria, increasing bacterial diversity and richness in pregnant women. A similar result was reported in another study of a Chinese cohort, whereby Huang et al. found higher vaginal bacterial diversity in pregnant women, especially in the first trimester [38]. A reasonable explanation might be that fluctuation in the vaginal microbiota differs between Asian populations and Western populations and is heavily influenced by ethnicity. Regardless, the intrinsic causes need to be further studied by investigations of larger scope.

HPV infection can increase vaginal bacterial richness and diversity and lower the percentage of *Lactobacillus* [11, 12], and our results are in agreement with these previous studies. HPV infection is thought to alter the acidic environment of the vagina, which might promote outbreaks of bacteria [12]. In addition, the mucosal immunity and inflammation induced by HPV infection, including induction of pro-inflammatory cytokines, production of reactive oxygen species and activation of immune cells, might lead to changes in the vaginal microbiota [39]. However, other studies have indicated that hrHPV is not necessarily sufficient to induce changes in the cervicovaginal microbiota [17, 40], even though enrichment of certain anaerobic bacteria was found in patients with CIN lesions [17]. As we did not conduct cervical biopsy for high-risk HPV-infected women in our study, the presence of CIN was not assessed. Hence, we cannot exclude the possibility that the vaginal microbiota was also influenced by CIN lesions [17]. Interestingly, we found that the influence of pregnancy on bacterial richness was greater than that of HPV infection. To date, Tuominen H. et al. has also reported an altered microbial composition in cervix and placenta of HPV positive pregnant women [14]. However, it remains uncertain whether the changes result from HPV or other factors. Although both pregnancy and hrHPV infection reduced the abundance of *Lactobacillus*, which may contribute to increased bacterial richness and diversity, it is clear that the underlying mechanisms responsible are different. Changes in physiological hormones and the immunosuppressive state that occurs during pregnancy might lead to a fragile balance of the vaginal microbiome, which should be further explored in future studies.

Regarding community state types, the most abundant CST among nonpregnant reproductive-age women (NPN) was CST III, which was in accordance with the results from an Asian population in the study by Ravel et al. [34]. The most dominant CST in the hrHPV infection and pregnancy groups (PN, PHR and NPHR) was CST I. The predominant CSTs during pregnancy reported in previous studies varied from CST III to CST I [19, 37, 41]. Several previous studies have shown that *L. iners* and *L. crispatus* are the two most abundant *Lactobacillus* species found in pregnant women [18, 19, 37, 38, 41–43]. In the vagina, a bacterial community change from CST III to CST IV is commonly observed, thus indicating that a colonization of anaerobes is more frequent in an *L. iners*-dominant VMB (vaginal microbiome). Conversely, an *L. crispatus-*dominant VMB has been associated with a low-stress environment and an adequate level of autophagy by vaginal epithelial cells to remove harmful cytoplasmic components, as well as bacteria, in pregnant women [41]. Similarly, the predominant CSTs in cases of HPV infection are controversial. Lee et al. reported that the prevalence of HPV infection did not differ between CST III and CST I [12], whereas another study found a higher HPV infection rate for an *L. iners*-dominant VMB [13]. It should be noted that these studies were performed using cohorts of different ethnicities, which might be one of the reasons for the differences. We also observed HPV infection to be associated with an increased proportion of CST IV in both nonpregnant and pregnant women. In two longitudinal studies, CST IV dominated by anaerobic bacteria comprised the greatest proportion of HPV-positive samples, and CST IV was associated with an increased risk of transitioning to an HPV-positive state [13, 44].

HPV infection and pregnancy equally influenced the vaginal microbial composition, but different specific genera were enriched in hrHPV-infected or pregnant women. *Bifidobacterium*, *Bacillus*, *Megasphaera*, *Sneathia*, *Prevotella*, *Gardnerella*, *Fastidiosipila* and *Dialister* were identified as significant taxa in nonpregnant hrHPV-infected populations; in previous studies, anaerobic bacteria such as *Bacillus*, *Megasphaera*, *Sneathia*, *Prevotella*, *Gardnerella* and *Dialister* have been associated with HPV infection [12, 13, 45, 46]. In general, a microenvironment with a high proportion of anaerobic bacteria and a lower proportion of *Lactobacillus* spp. is more susceptible to HPV infection. A surprising finding was that *Bifidobacterium*, a type of lactic acid-producing probiotic [47], was enriched in HPV-positive women. Although participants who used probiotics in the preceding 2 weeks were excluded from the study, we cannot rule out the influence of probiotics taken prior to that time, and they might have some persistent effects on the vaginal microbiota [48]. It has been hypothesized that *Bifidobacterium* might be able to guarantee a healthy vaginal balance by the production of lactic acid, in the lack of *Lactobacillus*. However, there are several cases reporting *Bifidobacterium* species as pathogens in various infectious conditions [49, 50], and a high level of stress inducers has been detected in vaginal epithelial cells when *Bifidobacterium* predominated the VMB [38]. Hence, the role of members of this genus should be further investigated. However, as both HPV infection and pregnancy are able to influence the vaginal microbiome, an inconsistent profiling of significant bacteria was found in hrHPV-infected pregnant women compared to nonpregnant women. Anaerobic bacteria (*Megasphaera* and *Bacillus*) and lactic acid-producing bacteria (*Bifidobacterium* and *Lactococcus*) were identified in these samples. Moreover, we found novel vaginal bacterial taxa, i.e., *Acidovorax* and *Oceanobacillus*, which were also associated with HPV infection in pregnant women. Nevertheless, the roles of these microorganisms are not clear and should be investigated further. These findings support our hypothesis that hrHPV infection and pregnancy have a dual effect on increasing vaginal bacterial diversity, leading to a differentially altered vaginal microbiota in HPV-infected pregnant women compared to nonpregnant HPV-infected women.

The strength of this study is that it addressed still-unresolved issues regarding the association between the VMB and HPV infection in pregnant women. We found high diversity and richness for the vaginal microbiome during pregnancy in a Chinese cohort and uncovered that pregnancy and HPV infection have a probable synergistic effect on altering the VMB, which caused a more complicated vaginal bacterial environment in pregnant women with hrHPV infection. The limitations of this study were that it was a small, cross-sectional study, and thus we could not determine any causation between the VMB and HPV infection or pregnancy. Further studies with longitudinal sampling are needed to assess correlations between the dynamics of the VMB microbiome and the transition of HPV at early, mild, and late gestational stages as well as postpartum and to investigate its relationship with different subtypes of HPV.

## Conclusion

This work uncovered a probable synergistic effect of hrHPV infection and pregnancy on the vaginal microbial composition. Despite not having measured hormone level in our patients, pregnancy status seems to be a bigger driver of the vaginal diversity in our cohort, as other studies demonstrated that HPV alone does not lead to changes in it [17, 40]. HPV infection in pregnant women was associated with a more complex and diverse microbial environment.

## Additional files

Additional file 1: **Table S1.** HPV genotyping and distribution in pregnant women. (DOCX 18 kb)

Additional file 2: **Table S2.** HPV genotyping and distribution in not pregnant women. (DOCX 17 kb)

Additional file 3: **Table S3.** Characteristics of study population. (DOCX 20 kb)

Additional file 4: **Table S4.** Sequences information. (DOCX 24 kb)

Additional file 5: **Figure S1.** Heat map of relative abundance for the 22 most abundant bacterial phyla found in the vaginal bacterial communities of 4 groups. (PDF 173 kb)

Additional file 6: **Figure S2.** Heat map of relative abundance for the 20 most abundant bacterial genus found in the vaginal bacterial communities of 4 groups. (PDF 142 kb)

Additional file 7: **Table S5.** The comparison of the bacterial structure between two groups tested by ANOSIM test. (DOCX 34 kb)

## Data Availability

All reads obtained were submitted to NCBI Sequence Read Archive (SRA) under the accession number SRP126438 (http://www.ncbi.nlm.nih.gov/bioproject/421398).

## Abbreviations

CIN: Cervical intraepithelial neoplasia
CST: Community state type
HPV: Human papillomavirus
hrHPV: High-risk human papillomavirus
LEfSe: Linear discriminant analysis (LDA) effect size
OTUs: Operational taxonomic units
PCoA: Principal coordinates analysis
RDA: Redundancy analysis
RDP: Ribosomal Database Project
TCT: ThinPrep cytology test
