# Supplementary material for: Association between the vaginal microbiome and high-risk human papillomavirus infection in pregnant Chinese women
Source: BMC Infect Dis. 2019 Aug 1;19:677. doi: 10.1186/s12879-019-4279-6 (PMC6669982; doi:10.1186/s12879-019-4279-6)
Supplement: Supplementary file 1 — Table S1. HPV genotyping and distribution in pregnant women. (DOCX 18 kb) [file 12879_2019_4279_MOESM1_ESM.docx]

**Table S1: HPV genotyping and distribution in pregnant women**

| **HPV genotype** | **N** | **(%)** |
| --- | --- | --- |
| 16 | 9 | 23.7 |
| 18 | 1 | 2.6 |
| 39 | 6 | 15.8 |
| 51 | 5 | 13.2 |
| 52 | 1 | 2.6 |
| 53 | 4 | 10.5 |
| 58 | 3 | 7.9 |
| 59 | 1 | 2.6 |
| 66 | 1 | 2.6 |
| 16/18 with other high-risk subtypes | 1 | 2.6 |
| Other high-risk subtypes mixed | 4 | 10.5 |
| With low-risk types | 2 | 5.3 |
