# Supplementary material for: Association between the vaginal microbiome and high-risk human papillomavirus infection in pregnant Chinese women
Source: BMC Infect Dis. 2019 Aug 1;19:677. doi: 10.1186/s12879-019-4279-6 (PMC6669982; doi:10.1186/s12879-019-4279-6)
Supplement: Supplementary file 2 — Table S2. HPV genotyping and distribution in not pregnant women. (DOCX 17 kb) [file 12879_2019_4279_MOESM2_ESM.docx]

# Table S2: HPV genotyping and distribution in not pregnant women

| **HPV genotype** | **N** | **(%)** |
| --- | --- | --- |
| 16 | 9 | 47.4 |
| 18 | 1 | 5.3 |
| 39 | 1 | 5.3 |
| 52 | 1 | 5.3 |
| 16/18 with other high-risk subtypes | 4 | 21.1 |
| With low-risk types | 3 | 15.8 |
