# Supplementary material for: Association between the vaginal microbiome and high-risk human papillomavirus infection in pregnant Chinese women
Source: BMC Infect Dis. 2019 Aug 1;19:677. doi: 10.1186/s12879-019-4279-6 (PMC6669982; doi:10.1186/s12879-019-4279-6)
Supplement: Supplementary file 3 — Table S3. Characteristics of study population. (DOCX 20 kb) [file 12879_2019_4279_MOESM3_ESM.docx]

# Table S3: Characteristics of study population

BMI: Body mass index; NA: not applicated; Quantitative data were shown as mean ± SD, one way ANOVA was used to compare the differences among four groups; Frequency data were shown as N(%), Fisher exact test was used to compare the differences among four groups; Bonferroni test was used to adjust P value between two groups; P<0.05 was significant, ^***^: P≤0.001；^*^P<0.05.

|  | **PHR (N=38)** | **PN (N=48)** | **NPHR (N=19)** | **NPN (N=30)** | **P value** |
| --- | --- | --- | --- | --- | --- |
| **Age（year）** | 30.13±4.79 | 29.77±3.43 | 33.53±3.64 | 34.90±4.17 | <0.001^***^ |
| **Gestational week (week)** | 24.80±3.63 | 26.60±1.69 | NA | NA | 0.015^*^ |
| **Education** |  |  |  |  | 0.920 |
| **Illiterate** | 0 (0) | 0 (0) | 0 (0) | 1 (3.3) |  |
| **Below high school** | 5 (23.8) | 11 (23.4) | 3 (27.3) | 7 (23.3) |  |
| **Bachelor or above** | 16 (76.2) | 36 (76.6) | 8 (72.7) | 22 (73.3) |  |
| **Smoking** |  |  |  |  | 0.260 |
| **Yes** | 6（28.6） | 7（14.6.） | 3（25.0） | 10（33.3） |  |
| **No** | 15 (71.4) | 41 (85.4) | 9 (75.0) | 20 (66.7) |  |
| **Vaginal douching** |  |  |  |  | 0.630 |
| **＞once per day** | 5 (25.0) | 9 (20.9) | 2 (16.7) | 3 (10.3) |  |
| **Once per day** | 13 (65.0) | 30 (69.8) | 10 (83.3) | 25 (86.2) |  |
| **＜once per day** | 2 (10.0) | 4 (9.3) | 0 (0) | 1 (3.4) |  |
| **BMI (kg/m^2^)** | 22.50±2.50 | 22.61±2.76 | 22.16±3.12 | 22.20±2.50 | 0.900 |
| **Sexual age (year)** | 23.14±4.96 | 23.04±2.83 | 22.42±3.23 | 23.21±2.38 | 0.916 |
| **Sexual partners** | 1.63±0.90 | 1.37±0.70 | 1.67±0.89 | 1.17±0.38 | 0.066 |
| **Gesity** | 1.85±1.04 | 1.83±1.14 | 2.42±1.31 | 2.03±0.96 | 0.387 |
| **Parity** | 0.25±0.44 | 0.25±0.44 | 1.33±0.65 | 1.17±0.53 | <0.001^***^ |
