# Supplementary material for: Association between the vaginal microbiome and high-risk human papillomavirus infection in pregnant Chinese women
Source: BMC Infect Dis. 2019 Aug 1;19:677. doi: 10.1186/s12879-019-4279-6 (PMC6669982; doi:10.1186/s12879-019-4279-6)
Supplement: Supplementary file 4 — Table S4. Sequences information. (DOCX 24 kb) [file 12879_2019_4279_MOESM4_ESM.docx]

**Table S4: Sequences information**

|  | **Sample ID** | **Reads Number** | **OTU Number** | **Average reads per group**  **(Mean ± SD)** | **Average OTUs per group**  **(Mean ± SD)** |
| --- | --- | --- | --- | --- | --- |
| **PN** | NP_18 | 29745 | 138 | 31211.96 ± 5716.34 | 98.02 ±33.33 |
|  | NP_41 | 27503 | 87 |  |  |
|  | NP_40 | 24633 | 105 |  |  |
|  | NP_42 | 27101 | 52 |  |  |
|  | NP_43 | 27422 | 47 |  |  |
|  | NP_44 | 33767 | 94 |  |  |
|  | NP_45 | 32408 | 94 |  |  |
|  | NP_46 | 20509 | 100 |  |  |
|  | NP_47 | 36048 | 94 |  |  |
|  | NP_48 | 37126 | 141 |  |  |
|  | NP_21 | 34388 | 99 |  |  |
|  | NP_29 | 23979 | 103 |  |  |
|  | NP_35 | 24885 | 167 |  |  |
|  | NP_34 | 22195 | 83 |  |  |
|  | NP_37 | 34209 | 113 |  |  |
|  | NP_36 | 28991 | 92 |  |  |
|  | NP_31 | 33104 | 107 |  |  |
|  | NP_30 | 33122 | 146 |  |  |
|  | NP_33 | 25862 | 97 |  |  |
|  | NP_39 | 36119 | 79 |  |  |
|  | NP_38 | 31113 | 88 |  |  |
|  | NP_32 | 39268 | 114 |  |  |
|  | NP_26 | 37167 | 146 |  |  |
|  | NP_27 | 33744 | 103 |  |  |
|  | NP_24 | 20308 | 129 |  |  |
|  | NP_25 | 36879 | 102 |  |  |
|  | NP_22 | 23858 | 120 |  |  |
|  | NP_20 | 20376 | 30 |  |  |
|  | NP_28 | 37747 | 129 |  |  |
|  | NP_23 | 29154 | 107 |  |  |
|  | NP_11 | 34124 | 129 |  |  |
|  | NP_10 | 27203 | 132 |  |  |
|  | NP_17 | 37075 | 129 |  |  |
|  | NP_16 | 38503 | 139 |  |  |
|  | NP_15 | 36886 | 118 |  |  |
|  | NP_14 | 27596 | 119 |  |  |
|  | NP_19 | 33598 | 92 |  |  |
|  | NP_7 | 36913 | 65 |  |  |
|  | NP_6 | 37609 | 47 |  |  |
|  | NP_5 | 32550 | 68 |  |  |
|  | NP_4 | 29302 | 30 |  |  |
|  | NP_3 | 29892 | 46 |  |  |
|  | NP_2 | 39753 | 46 |  |  |
|  | NP_1 | 22189 | 67 |  |  |
|  | NP_9 | 39741 | 35 |  |  |
|  | NP_8 | 37033 | 111 |  |  |
|  | NP_13 | 28706 | 123 |  |  |
|  | NP_12 | 26771 | 103 |  |  |
| **NPHR** | 59_ZQ | 21395 | 153 | 29962.00 ±7467.15 | 70.53 ± 40.35 |
|  | 52_CYH | 27431 | 86 |  |  |
|  | 43_ZLY | 27675 | 69 |  |  |
|  | 63_WHX | 27110 | 87 |  |  |
|  | 200_CHY | 23677 | 115 |  |  |
|  | 16_CXH | 23154 | 88 |  |  |
|  | 80_FL | 23677 | 115 |  |  |
|  | 77_HQ | 32509 | 74 |  |  |
|  | 83_DLY | 26624 | 139 |  |  |
|  | 20_TWJ | 39319 | 62 |  |  |
|  | 73_JZY | 39959 | 84 |  |  |
|  | YQM | 39113 | 67 |  |  |
|  | YXX | 43807 | 36 |  |  |
|  | DMH | 41468 | 34 |  |  |
|  | 385_ZLQ | 30610 | 18 |  |  |
|  | 331_QY | 21331 | 24 |  |  |
|  | 374_BXR | 32852 | 44 |  |  |
|  | 343_XHQ | 26292 | 22 |  |  |
|  | 318_LCQ | 21275 | 23 |  |  |
| **NPN** | 4389 | 23436 | 17 | 26130.77 ± 4375.90 | 22.47 ±9.50 |
|  | 4383 | 23778 | 15 |  |  |
|  | 4387 | 20807 | 39 |  |  |
|  | 4374 | 25158 | 17 |  |  |
|  | 4409 | 26411 | 33 |  |  |
|  | 4404 | 26976 | 21 |  |  |
|  | 4419 | 32863 | 37 |  |  |
|  | 4417 | 26866 | 16 |  |  |
|  | 4411 | 29198 | 27 |  |  |
|  | 4341 | 33798 | 37 |  |  |
|  | 4351 | 23824 | 13 |  |  |
|  | 4366 | 32050 | 16 |  |  |
|  | 4349 | 26886 | 23 |  |  |
|  | 4348 | 34063 | 17 |  |  |
|  | 4347 | 29494 | 12 |  |  |
|  | 4343 | 20668 | 46 |  |  |
|  | 4340 | 23329 | 14 |  |  |
|  | 4399 | 21373 | 22 |  |  |
|  | 4375 | 28138 | 16 |  |  |
|  | 4391 | 26750 | 12 |  |  |
|  | 4360 | 21131 | 13 |  |  |
|  | 4362 | 23564 | 38 |  |  |
|  | 4369 | 20932 | 23 |  |  |
|  | 4368 | 32014 | 22 |  |  |
|  | 4398 | 32921 | 10 |  |  |
|  | 4390 | 21922 | 18 |  |  |
|  | 4392 | 21365 | 30 |  |  |
|  | 4393 | 23045 | 20 |  |  |
|  | 4394 | 29362 | 24 |  |  |
|  | 4395 | 21801 | 26 |  |  |
| **PHR** | 109_LL | 35198 | 106 | 29978.45 ± 5330.48 | 116.79 ± 37.57 |
|  | 111_XBY | 35530 | 85 |  |  |
|  | 103_SXF | 29274 | 105 |  |  |
|  | 187_XLN | 24131 | 153 |  |  |
|  | 101_ZJ | 24540 | 97 |  |  |
|  | 175_WZC | 27193 | 81 |  |  |
|  | 7_WY | 28522 | 139 |  |  |
|  | 181_MQ | 31921 | 161 |  |  |
|  | 136_ZY | 27209 | 126 |  |  |
|  | 2_KLJ | 27179 | 125 |  |  |
|  | 197_WZC | 24211 | 48 |  |  |
|  | 5_LLQ | 38664 | 102 |  |  |
|  | 144_LM | 27261 | 114 |  |  |
|  | 6_DS | 22638 | 141 |  |  |
|  | 4_LYX | 29929 | 93 |  |  |
|  | 119_HW | 24913 | 85 |  |  |
|  | 8_ZP | 32232 | 180 |  |  |
|  | 100_TL | 35714 | 88 |  |  |
|  | 172_YN | 31558 | 193 |  |  |
|  | 108_LCL | 23295 | 47 |  |  |
|  | 177_DY | 38423 | 163 |  |  |
|  | 166_YQ | 30690 | 159 |  |  |
|  | 131_ZX | 30520 | 118 |  |  |
|  | 104_DSY | 38946 | 135 |  |  |
|  | 110_MYM | 30641 | 82 |  |  |
|  | 98_ZY | 31634 | 93 |  |  |
|  | 135_YJL | 24988 | 113 |  |  |
|  | 19_YLL | 28115 | 90 |  |  |
|  | 127_HJM | 38557 | 100 |  |  |
|  | 142_HH | 23298 | 199 |  |  |
|  | 158_LYT | 24924 | 146 |  |  |
|  | 165_LX | 26127 | 132 |  |  |
|  | 151_CAQR | 32937 | 172 |  |  |
|  | 120_LHY | 25387 | 74 |  |  |
|  | 147_LMY | 34350 | 130 |  |  |
|  | 112_YL | 21317 | 74 |  |  |
|  | 107_LXH | 39928 | 94 |  |  |
|  | 102_NYM | 37287 | 95 |  |  |
