# Supplementary material for: Association between the vaginal microbiome and high-risk human papillomavirus infection in pregnant Chinese women
Source: BMC Infect Dis. 2019 Aug 1;19:677. doi: 10.1186/s12879-019-4279-6 (PMC6669982; doi:10.1186/s12879-019-4279-6)

Figure S1: Heat map of relative abundance for the 22 most abundant bacterial phyla found in the vaginal bacterial communities of 4 groups

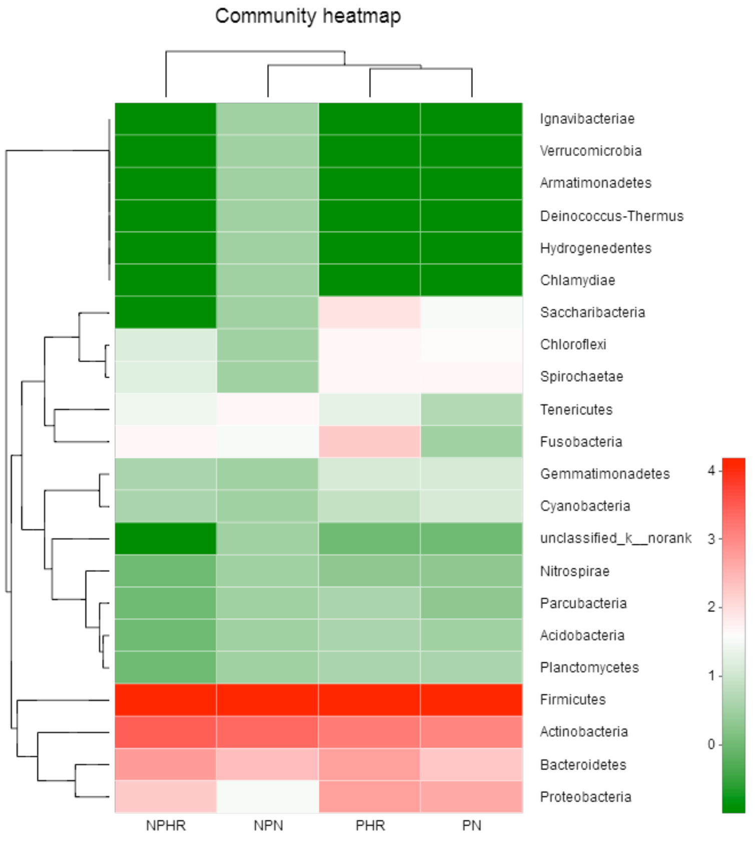

Supplement: Supplementary file 5 — Figure S1. Heat map of relative abundance for the 22 most abundant bacterial phyla found in the vaginal bacterial communities of 4 groups. (PDF 173 kb) [file 12879_2019_4279_MOESM5_ESM.pdf]
