# Supplementary material for: Association between the vaginal microbiome and high-risk human papillomavirus infection in pregnant Chinese women
Source: BMC Infect Dis. 2019 Aug 1;19:677. doi: 10.1186/s12879-019-4279-6 (PMC6669982; doi:10.1186/s12879-019-4279-6)
Supplement: Supplementary file 7 — Table S5. The comparison of the bacterial structure between two groups tested by ANOSIM test. (DOCX 34 kb) [file 12879_2019_4279_MOESM7_ESM.docx]

**Table S5: The comparison between two groups tested by ANOSIM test**

| Group | R value | P value |
| --- | --- | --- |
| PHR vs, NPHR | 0.3232 | 0.001 |
| PHR vs. NPN | 0.8203 | 0.001 |
| NPHR vs. PN | 0.2871 | 0.001 |
| NPHR vs. NPN | 0.3881 | 0.001 |
| PN vs. NPN | 0.8043 | 0.001 |
| PHR vs. PN | -0.0062 | 0.545 |
